# Chancre mou

**DOI:** 10.11604/pamj.2019.33.185.16187

**Published:** 2019-07-11

**Authors:** Fatima-Zahra Agharbi

**Affiliations:** 1 Centre Hospitalier Régional Tétouan, Tétouan, Maroc

**Keywords:** Ulcération, chancre mou, azithromycine, Ulceration, chancroid, azithromycin therapy

## Abstract

Chancroid (also known as soft chancre and ulcus molle) is a sexually transmitted disease (STD) due to the Ducrey’s bacillus (or Haemophilus ducreyi) characterized by chancre at the site of ulcerated inoculation associated with lymphadenopathy. The disease manifests as a small pinkish papule at the site of penetration of the bacterium. After an incubation period ranging from 24 hours to 15 days (on average 5 days). The lesion rapidly evolves into a more or less extended pinkish, painful, deep ulcer with very inflamed and sharp edges and a ragged appearance. The lymphadenopathies usually occur 2-3 weeks after the contact. They are often unilateral and can evolve into ulcers with pus discharge at the level of the skin. Some complications can occur: penile gangrene, extended gangrene of the skin, local superinfection, association with other sexually transmitted diseases. Bacterium can be identified by microscopic examination of a smear of the chancre-like ulcer, more rarely by fine-needle puncture biopsy of a lymphadenopathy. Giemsa or Pappenheim coloration allows identification of the germ. Treatment is based on azithromycin (1 g per os in a single dose) or ceftriaxone (250 mg administered intramuscularly in a single dose). We report the case of a 30-year old man with well-defined deep scrotum ulcer with necrotic center which occurred 1 week after unprotected sexual intercourse. Haemophilus ducrey has been detected by culture and the patient underwent Azithromycin therapy with good outcome.

## Image en médecine

Le chancre mou (ou chancrelle ou chancre de Ducrey) est une maladie sexuellement transmissible (MST) due au bacille de Ducrey (ou Haemophilus ducreyi) caractérisée par un chancre d'inoculation ulcéré associée à des adénopathies. La maladie se manifeste, après une période d'incubation variant de 24 heures à 15 jours (en moyenne 5 jours), par une petite papule rosée au lieu de pénétration de la bactérie. La lésion évolue rapidement vers une ulcération plus ou moins étendue, rosée, douloureuse, profonde, aux bords très inflammatoires et nets, d'aspect déchiqueté. Les adénopathies, apparaissant 2 à 3 semaines après le contact. Elles sont souvent unilatérales, et peuvent évoluer vers l'ulcération avec écoulement de pus au niveau de la peau. Des complications sont possibles: gangrène de la verge, gangrène cutanée étendue, surinfection locale, association à d'autres IST. L'identification de la bactérie peut se faire par examen microscopique d'un frottis du chancre, plus rarement par ponction à l'aiguille fine d'une adénopathie. La coloration de Giemsa ou celle de Pappenheim permet d'identifier le germe. Le traitement fait appel à l'azithromycine (1 g per os en dose unique) ou à la ceftriaxone (250 mg par voie intramusculaire en dose unique). Nous rapportons l'observation d'un homme de 30 ans qui présentait 1 semaine après un rapport sexuel non protégé une ulcération du scrotum bien limitée profonde à centre nécrotique. L'haemophilus ducrey a été mis en évidence à la culture et le patient a été mis sous approche Azithromycine avec bonne évolution.

**Figure 1:**
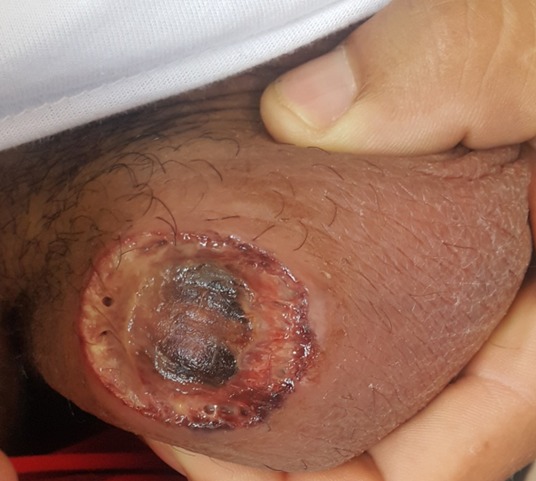
Ulcération du scrotum bien limitée profonde à centre nécrotique

